# Jurassic paleosurfaces with fecal mounds reveal the last supper of arenicolid worms

**DOI:** 10.1038/s41598-023-51103-2

**Published:** 2024-01-06

**Authors:** M. Gabriela Mángano, Luis A. Buatois, Laura Piñuela, Nils Volkenborn, Francisco J. Rodríguez-Tovar, José C. García-Ramos

**Affiliations:** 1 Department of Geological Sciences, University of Saskatchewan, 114 Science Place, Saskatoon, SK S7N 5E2 Canada; 2 Museo del Jurásico de Asturias (MUJA), 33328 Colunga, Asturias Spain; 3 School of Marine and Atmospheric Sciences, Stony Brook University, Stony Brook, NY 11794 USA; 4 Departamento de Estratigrafía y Paleontología, Universidad de Granada, 18002 Granada, Spain

**Keywords:** Ecology, Palaeontology, Sedimentology

## Abstract

Exceptional paleosurfaces preserving fecal casting mounds occur in the Upper Jurassic Lastres Formation of Spain. As in modern shorelines, these biogenic structures are associated with straight to sinuous-crested ripples showing the interplay of biological and physical processes in a low-energy marine environment. These trace fossils display characteristics, distribution, and densities like those of modern arenicolid populations (approximately 35 specimens per m^2^). Under close examination, these fecal casting mounds are morphologically undistinguishable from those produced by recent arenicolids (e.g. *Arenicola marina, Abarenicola pacifica*), providing evidence of the presence of these polychaetes in the Late Jurassic. As their modern counterparts, fossil arenicolids very likely modified their environment generating a seabed topography and impacting ancient benthic communities, sediment characteristics, and sediment biogeochemistry. Although the presence of oxic microhabitats and biogeochemical processes cannot be accurately measured in the fossil record, comparison with the work of modern populations allows to make inferences on sediment reworking and bioirrigation potential. In addition, association with grazing trails supports the idea of fertilization and modulation of food resources to other species. These paleosurfaces underscore the significance of high-fidelity snapshots in the fossil record (true substrates) to reconstruct past ecologies and sediment biogeochemistry. A new ichnotaxon, *Cumulusichnus asturiensis* n. igen. and n. isp., is defined.

## Introduction

Bioturbation includes all transport processes, including movement of both sediment and fluids, generated by the activity of animals that directly or indirectly affect sediment fabric and enhance benthic pelagic coupling^1^. A wide diversity of behaviors ranging from bulldozing of the surficial sediment layer to vertical sediment mixing and bioirrigation of deep-reaching burrows result in modifications of the seabed^1,2^. Bioturbation plays a critical role for organic matter mineralization^3^ and in early diagenesis^4^, and had significant impacts on ocean and atmosphere chemistry on geological time scales^5,6^. In this paper, we document the exceptional preservation of an ancient biogenically modified seabed characterized by the presence of coiled fecal mounds associated with vertical burrows in the subaqueous delta plain of the Kimmeridgian (Upper Jurassic, 163.5–145 Ma) Lastres Formation of Asturias, northern Spain (Fig. S1). These biogenic structures are indistinguishable from those produced by lugworms (e.g., *Arenicola marina*) in modern tidal flats. The spatial and temporal occurrence of Jurassic fecal mounds—in combination with well-documented impacts of lugworm bioturbation on the structure and function of intertidal flat ecosystems today—suggest that these polychaetes likely played a significant role as ecosystem engineers in Jurassic shoreline settings. This article (1) documents a biogenically modified Jurassic seabed from the perspective of true substrates, as a direct window to Jurassic benthic ecology, (2) explores the potential factors controlling its preservation, (3) discusses its significance in terms of the macroevolutionary record of ecosystem engineering, and (4) defines a new ichnotaxon to name these distinct trace fossils.

## Stratigraphic and paleoenvironmental setting

**Figure 1:**
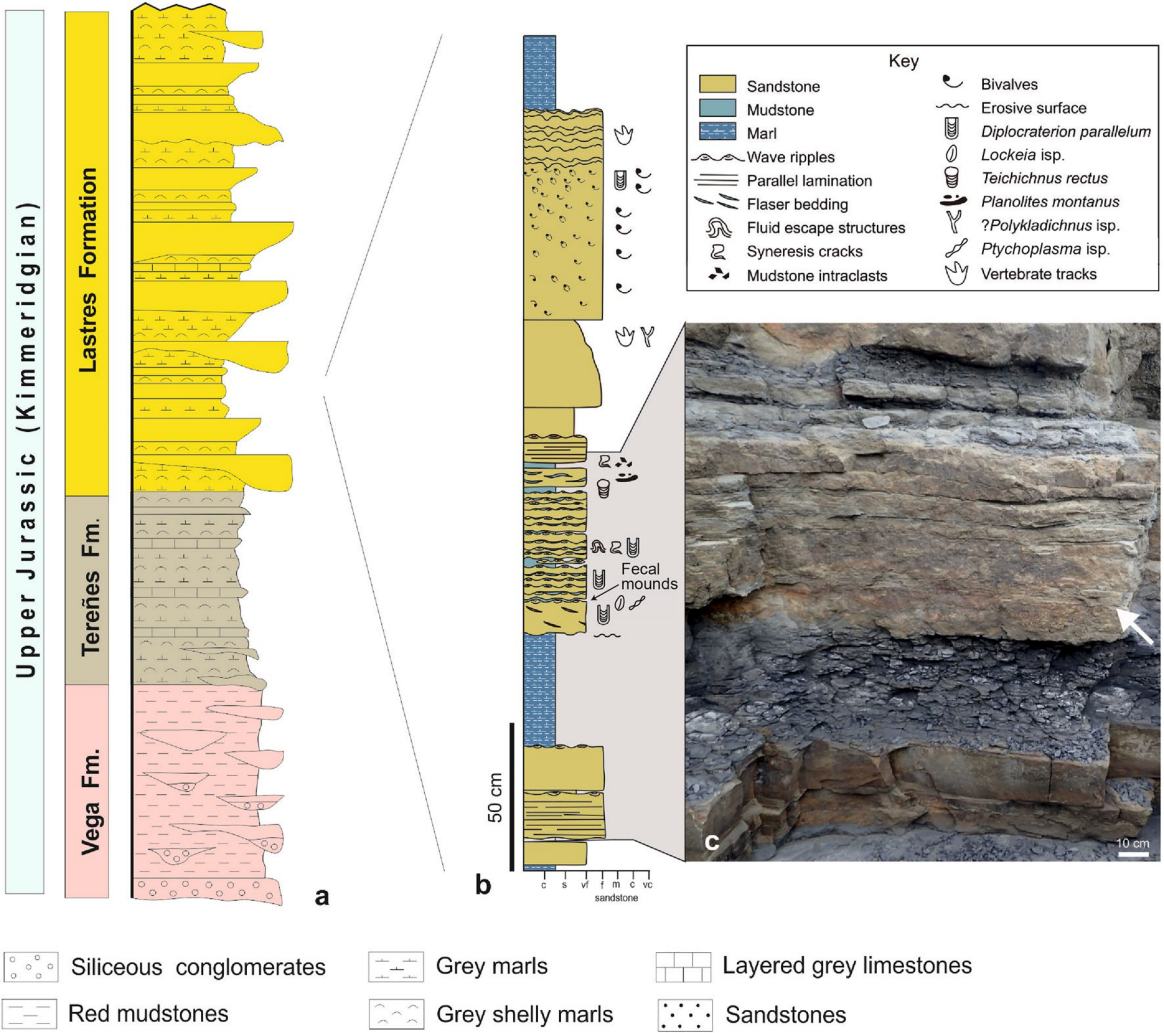
Stratigraphy and sedimentologic characteristics of the deposits. (**a**) General stratigraphy of the studied area. The lowermost Upper Jurassic unit, the La Ñora Formation, is not exposed in this area. (**b**) Detailed sedimentologic log of the interval of the Lastres Formation at Arroyo Solero containing one of the surfaces with fecal cast mounds, drawn by Maximiliano Paz using Adobe Illustrator CS6 version 16. (**c**) Close-up of the deposits showing in cross section the position of the bedding surface that contains fecal cast mounds (arrowed).

The Upper Jurassic of Asturias, northern Spain, crops out for 57 km along the coast from Gijon (in the west) to Ribadesella (in the east) (Fig. S1). The region is known as “The Dinosaur Coast”, famous worldwide for its dinosaur footprints and trackways, as well as for those of pterosaurs, turtles, and crocodiles^7–15^. The Upper Jurassic succession is divided into four formations overall, in ascending order: La Ñora, Vega, Tereñes, and Lastres (Fig. 1). The Lastres Formation consists of about 400 m of grey sandstone, mudstone, and marl, locally with conglomerate layers, recording sedimentation in a river-dominated delta that prograded into a restricted basin (shelf lagoon), protected from intense hydrodynamic forces^8,16,17^. Deltaic sedimentation was repeatedly interrupted by short-term transgressions, which are recorded by laterally extensive bivalve and gastropod shell beds. The fecal cast mounds were found in situ in Arroyo Solero (Figs. S1, 1b,c), as well as on isolated blocks belonging to the same outcrop belt of the Lastres Formation east of Playa España, both sections near the town of Villaviciosa (Fig. S1).

In Arroyo Solero, the fecal cast mounds occur on a 12.0 cm thick, flaser-bedded, poorly sorted, medium- to very fine-grained sandstone bed covered by a 0.1 cm mudstone parting (Fig. 1c). *Diplocraterion parallelum* (Fig. S2h,i)*,* small *Lockeia* isp. and *Ptychoplasma* isp., and undetermined simple grazing trails are also present in this bed. The trace fossil-bearing sandstone occurs within a sandstone-dominated heterolithic succession interbedded with shell beds (Fig. 1b). Wave-ripple cross-lamination, parallel lamination, flaser bedding, and syneresis cracks are the dominant sedimentary structures in this interval (Fig. 1b). In addition to *Diplocraterion parallelum* and the grazing trails, other beds in the succession contain *Teichichnus rectus*, *Planolites montanus*, and possible *Polykladichnus* isp. This stratigraphic interval of the Lastres Formation is interpreted as deposited in protected, brackish-water, low-energy bays of the subaqueous delta plain.

**Figure 2:**
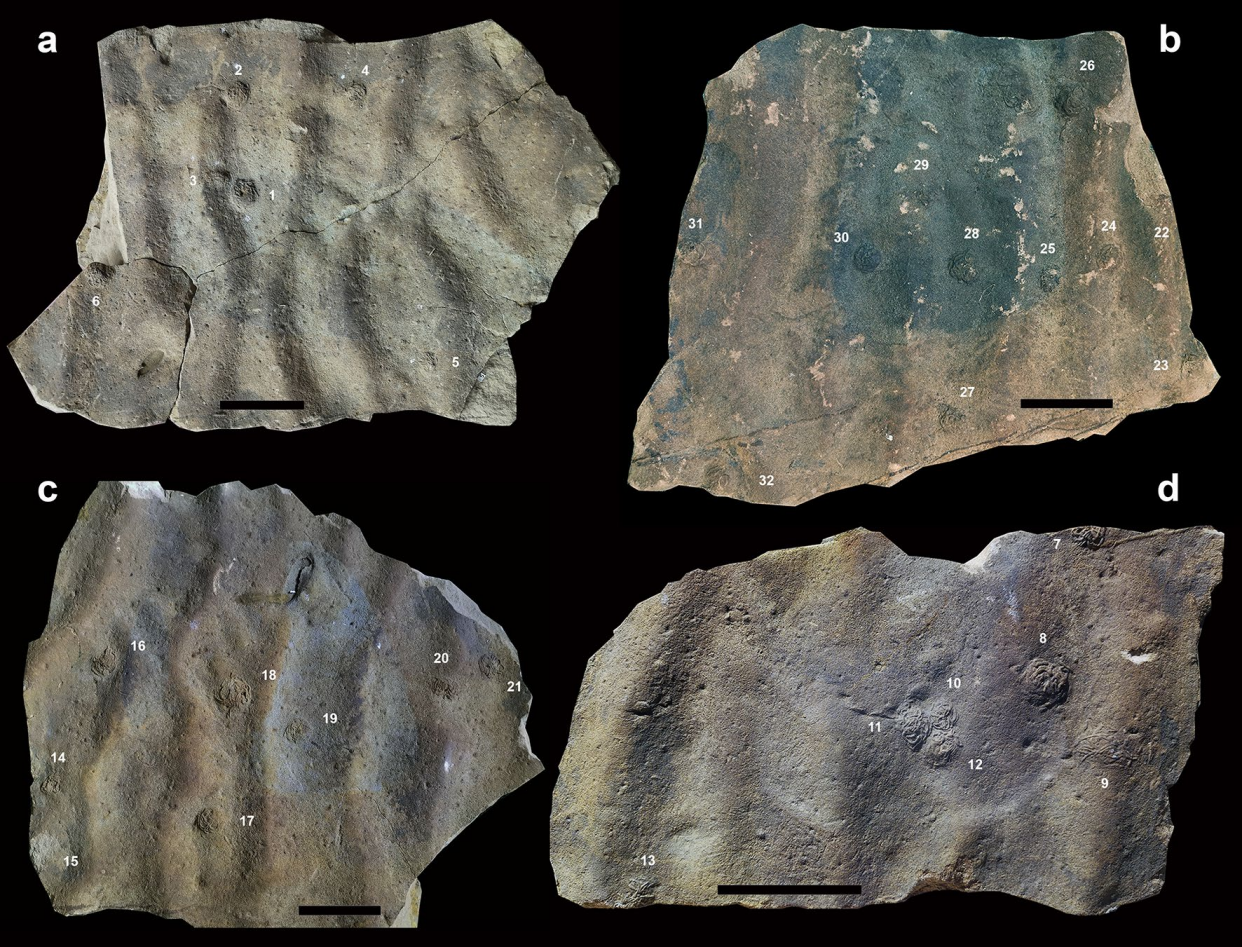
General views of samples with fossil arenicolid mounds from Playa España (Villaviciosa), Upper Jurassic Lastres Formation of Asturias, Spain. (**a**) MUJA-3596. (**b**) MUJA-3595. (**c**) MUJA-3594. (**d**) MUJA-3826. All scale bars are 10 cm long.

In Playa España, the fecal cast mounds occur on loose blocks of 4.0–6.5 cm thick, poorly sorted, medium- to very fine-grained sandstone beds displaying a subtle normal grading and straight to sinuous-crested ripples at the top (Fig. 2). Although the precise horizon from where these blocks are coming cannot be determined, the succession in this locality consists of alternations of sandstone, mudstone, and marl. The only associated trace fossils are tiny grazing trails assigned to *Archaeonassa fossulata* (Fig. S2c) and small (0.8–1.5 mm), up to 42 mm deep, sandy mud-filled, vertical to subvertical burrows usually seen as very small circular holes on sandstone tops (Fig. S2b) displaying diverse morphologies in cross section (Fig. S2e–g).

## Occurrence and main characteristics of the mounds

**Figure 3:**
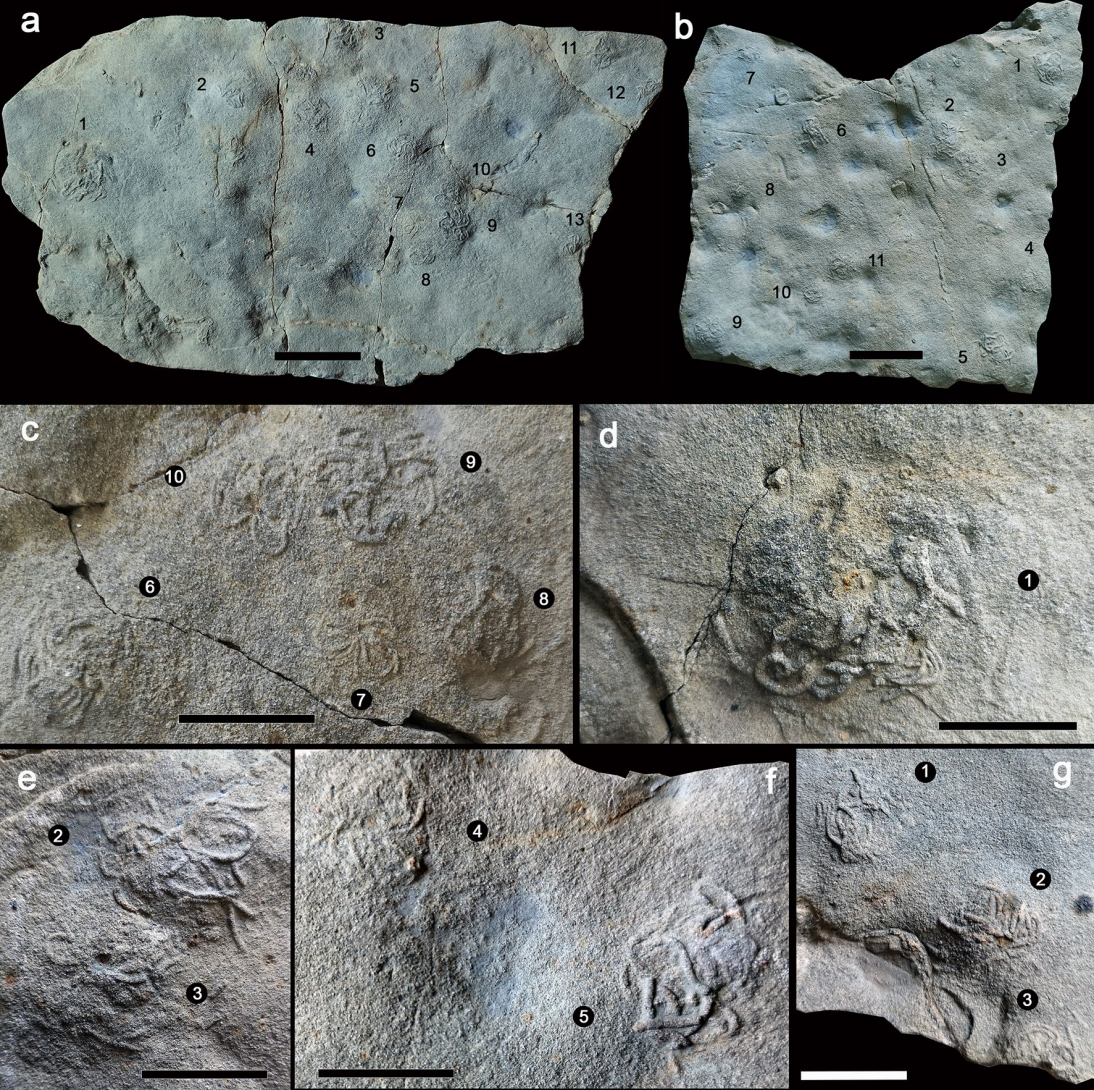
Fossil examples of arenicolid mounds from Arroyo Solero (Villaviciosa), Upper Jurassic Lastres Formation of Asturias, Spain. (**a**,**b**) General views of MUJA-4612 and MUJA-4613, respectively. (**c**,**d**) Details of some specimens of MUJA-4612. (**e**,**f**) Details of some specimens of MUJA-4613. (**g**) Details of some specimens of MUJA-4614. Note the center burrow opening in specimens in (**c**) (specimen 10), in (**d**), in (**e**) (specimen 3), in (**f**) (specimen 4), and in (**g**) (specimens 1 and 2). Scale bars are 5 cm long in (**a,b**); the rest of scales are 2 cm long.

**Figure 4:**
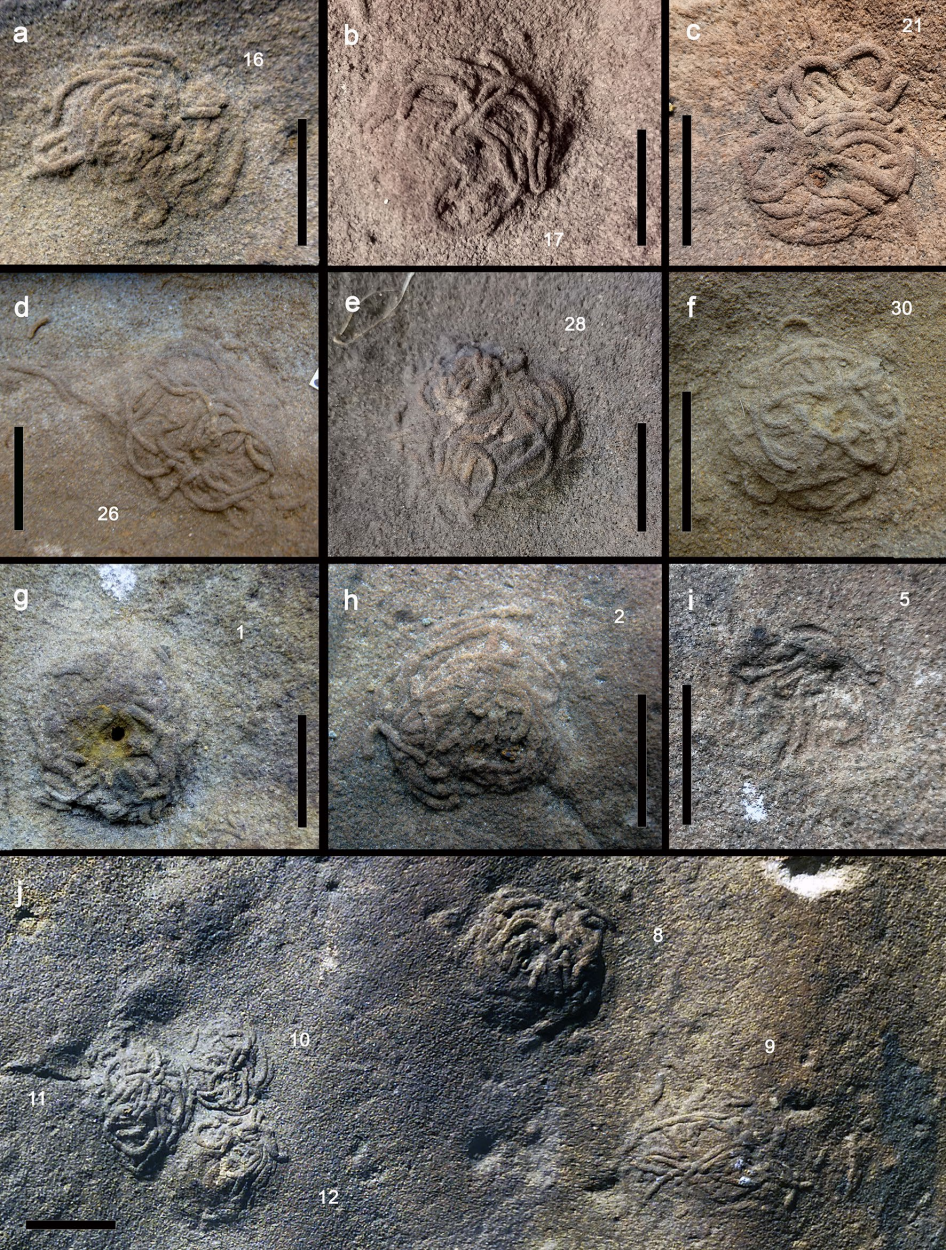
Fossil examples of arenicolid fecal mounds from Playa España, Upper Jurassic Lastres Formation of Asturias, Spain. (**a**–**c**) MUJA-3594-16, 17 (paratype) and 21 (holotype), respectively. (**d**–**f**) MUJA-3595-26, 28 (paratype) and 30, respectively. (**g**–**i**) MUJA-3596-1, 2 (paratype) and 5, respectively. (**j**) MUJA-3826-8, 9, and 10 and 11 (paratypes). Observe the central burrow opening in (**b,f,g,i**) (specimen 8) and off-center burrow opening in (**c,d**). Note the larger size compares to the specimens from Arroyo Solero. Scale bars are 2 cm long.

The biogenic structures are preserved as full reliefs and consist of a vertical to inclined burrow associated to a conspicuous flattened semispherical mound typically covered by coiled fecal strings defining a distinctive pit-and-mound topography on the sandstone top (Figs. 2, 3, 4). A new ichnotaxon, *Cumulusichnus asturiensis* n. igen. and n. isp., is defined for these trace fossils (see Appendix).

The best-preserved specimens come from Playa España, where mounds are up to 6 mm in height (Table S1). Diameter of the mounds is 14.9–42.4 mm; fecal string diameters are 0.9–2.3 mm. In some cases, a central or off-center, iron-stained burrow opening (1.6–3.9 mm wide) is clearly visible (Fig. 4c,d,g). Mounds define a conspicuous topography on surfaces characterized by straight to sinuous-crested oscillatory ripples. Specimens are dominantly present in ripple troughs (71%), with secondary distribution in ripple slopes (23%), and very rarely on ripple crests (6%). Discrete mounds are formed by intertwined spaghetti-like, coiled fecal strings. A few specimens show a fan-like or radiating distribution of castings connected to an apical or displaced mound depression (i.e. inferred vertical burrow opening) (Fig. 4d; j specimen 9). Others are represented by a smooth mound only locally covered by fecal castings (Fig. 4i). Fecal strings are cylindrical to subcylindrical and separate easily from the host rock suggesting they were strongly mucus lined. Individual mounds may be composed of more than a single diameter of fecal castings. Densities of up to 35 mounds per m^2^ have been recorded.

Specimens from Arroyo Solero are not as well preserved and differ slightly in size range (Table S2), but essentially record the same morphology and density. In this locality, mounds are up to 2 mm high and between 9.7–38.4 mm wide. Fecal string diameter is 0.7–1.6 mm. Similar central or off-center, iron-stained burrow openings (1.0–3.8 mm wide) associated with fecal cast mounds present in Playa España occur in Arroyo Solero specimens. The surface is irregular with no clear ripple trains. Specimens consist of fecal strings radiating irregularly from the top of the mound or from the burrow opening, in some cases becoming distally curved (Fig. 3c).

## Discussion

### Identifying the tracemakers

Arguably the most impressive biogenically generated seascapes in low-energy modern littoral settings are produced by dense populations of three groups of organisms: arenicolid polychaetes (e.g. *Arenicola marina*, *Abarenicola pacifica*), thalassinid shrimps (e.g. *Neotrypaea californiensis*, *Callianassa truncata*, *Glypturus acanthochirus*), and enteropneust hemichordates (e.g. *Schizocardium*, *Balanoglossus*). Thalassinids are known to produce relatively large (in some cases up to 1 m wide) mound structures, which generate a distinctive topography^18–21^. However, thalassinids typically produce loose aggregations of fecal pellets or pile up excavated sediment in mounds around burrow openings that, although somewhat resilient to physical reworking^20^, are easily dispersed by currents. Moreover, the feces typically consist of short, cylindrical fecal pellets that differ significantly from arenicolids’ longer, spaghetti-like fecal castings. Enteropneusts are also common modifiers of the seabed surface, producing fecal castings similar at first sight to those of arenicolids. However, shallow-water, acorn worm castings are formed of unconsolidated sand with high water content, resulting in softer castings with low preservational potential. On the contrary, fecal castings of arenicolids consist of densely packed sand forming cohesive thick, mucus-lined, fecal strings that are ejected through the tail shaft opening onto the sediment surface. Typically, about 1–2 mL of sediment are ejected approximately every 30 min and fecal coils can accumulate over many days which results in the formation of conspicuous fecal mounds. Fecal mounds of *Arenicola marina* are commonly smoothed and dispersed by tidal currents and wave action, resulting in characteristic flattened mounds on top of which new defecated material is accumulated. Some of the Lastres Formation specimens display this well-developed mound morphology, partially covered with “fresh”, well-preserved feces. Because the tail shaft is the only part of the lugworm burrow lined with mucus, it has higher preservability and is commonly the only part of the burrow that fossilizes. The associated small, mud-filled, vertical to subvertical burrows may have been tail shafts of juvenile arenicolids, which create similar burrows as adult lugworms but with a smaller burrow diameter. However, vertical burrows without a fecal mound (above the tail shaft) or a depression (above the head shaft) may have been created by other polychaetes. Intertidal flats with dense lugworm populations today are inhabited by other, mostly smaller, worm-like taxa with a wide range of quite variable burrow morphologies, and it seems plausible that the Jurassic seabed was the habitat of other organisms. The difficulties in assigning a vertical burrow structure to a specific ichnotaxa without information about the surface microtopography highlights the significance of these exceptionally well-preserved paleosurfaces in the Upper Jurassic Lastres Formation of Spain.

Jurassic mounds formed by accumulation of fecal castings connected to vertical burrows reveal striking similarities with modern structures produced by arenicolid polychaetes^22–24^ (Figs. 2a–d, 3a–g, 4a–j, S2c, S3a–h). Lugworms live head down in J-shaped, mucus-lined blind-ending burrows^24–27^. Surface sediment is subducted through the vertical head shaft down to the feeding pocket and ingested by the worm at 10–30 cm depth. Particles > 1 mm are rejected, which often leads to the accumulation of a shell debris below the depth of the feeding pockets^28,29^. Sediment subduction leads to the formation of conical depressions on the surface, often referred to as feeding funnels^23,26^. The distance between such depressions and the fecal cast mounds typically approximates the body length of the worm. For respiration, arenicolids ventilate their burrows by pumping water in a tail-to-head direction, injecting oxygenated water into the surrounding sediment inducing porewater advection in the surrounding sediment^24^. A similar function is inferred for ancient arenicolids, involving irrigation leading to profound changes in benthic habitat in Jurassic coastal settings^30^. With 30 individuals per m^2^, the upper 15 cm of the sediment pass through the lugworms once per year^31^ and approximately 2.7 L of oxic water are injected into the sediment per sqm and hour^32^, leading to complete porewater turnover of a few days in such densely populated sediments^26^. Over the entire European Wadden Sea and assuming lugworm densities of 17 individuals per m^2^, a 6 to 7 cm thick layer is reworked during a year^33^. In the analyzed fossil surfaces, up to 35 fecal mounds per m^2^ suggest an active lugworm population akin to moderate to high density populations^34,35^. A direct comparison with modern populations of similar densities suggests that Jurassic arenicolids may have pumped at rates of 3 L per hour per square meter, reworking the uppermost centimeter of sediment up to 15 times per year.

The patchy fossil record of annelids in general and of polychaetes in particular, mostly restricted to deposits of exceptional preservation, has complicated reconstruction of their evolutionary history^36^. Early claims of arenicolid body fossils in the Triassic^37,38^ are now considered doubtful^36^. Accordingly, the body-fossil record of arenicolids is essentially non-existent. The occurrence of these modified paleosurfaces hosting the in situ constructions produced by the fecal casts of arenicolids represents the first evidence of this group of polychaetes in the fossil record. In addition, comparisons with modern densities indicate that biogenic modification and ecosystem engineering by these organisms of low energy shorelines can be at least tracked to the Late Jurassic. Previous recordings of other vertical burrows (e.g. *Arenicolites*) cannot be regarded as evidence of arenicolids as these biogenic structures can be produced by different groups of organisms.

### Ecosystem engineering in the fossil record

The concept of ecosystem engineering refers to the modification of the environment by the action of organisms that directly or indirectly modulate the availability of resources to other species^39,40^. A remarkable example of allogenic ecosystem engineering in coastal environments is the remodelling of sedimentary surfaces and habitats by the burrowing activities of animals^2^. One of the earliest studies on the role of bioturbation in ecosystem engineering was Darwin^41^’s pioneering study on the effects of earthworm bioturbation on soil formation^2,42^.

Secular increases in trace-fossil diversity, bioturbation intensity and burrowing depth paralleled a steady increase in the importance of ecosystem engineering during the Phanerozoic. Regardless the complexity in terms of ultimate causes and feedback loops, the Cambrian explosion itself was likely driven by ecosystem engineering^43–45^. In particular, the Cambrian Agronomic Revolution marks a major shift in benthic ecology and community structure^46^. The establishment of a deep suspension-feeder infauna recorded by the appearance of *Skolithos* piperocks and other ichnofabrics dominated by vertical burrows^47,48^ may have triggered a pulse of diversification of detritus and deposit feeders during the second half of the early Cambrian^44^. The high densities of bioturbation in these ichnofabrics resulted in more efficient oxygen supply to deep part of the sediment as well as efficient flushing out or oxygenation of toxic porewater constituents, such as H_2_S^49–51^ creating new habitats for other infaunal organisms, engineering the infaunal ecospace, and increasing the complexity of the trophic web^44,52^. However, only with the rise of Modern Evolutionary Fauna during the Mesozoic Marine Revolution^53^ ecosystem engineering likely reached levels similar to modern coastal environments. By the Late Jurassic the revolution was fully underway, with crustaceans, modern bivalves, echinoids, and a wide variety of worms, particularly polychaetes, already playing a dominant role as bioturbators^54^.

Ecosystem engineering by modern arenicolid polychaetes operates on various spatial and temporal scales. Burrowing and pumping activity of modern lugworms results in well documented effects on the environment including the modification of the depositional surface, and changes in sediment grain size distribution and composition^55^, sediment permeability^30^, biogeochemical processes^56^, and benthic communities^57,58^. It is plausible that Jurassic arenicolids had similar impacts. The lugworm burrow itself provides diverse subsurface oxic microhabitats that are exploited by small sediment infauna, including copepods, amphipods, turbellarians, oligochaetes, nemertines, and small polychaetes^59,60^. At the sediment surface, the depressions accumulate organic material^61^ and become small water ponds that serve as a pit for copepods, turbellarians, and juvenile shrimps during low tide^62^. The irrigation activity and the related oxygen supply stimulate growth of digestible microorganisms in the feeding pocket, a concept that has been referred as gardening^63^. As a counterpart, bioadvection of nutrient-rich porewater from depth can fertilize microphytobenthic growth at the sediment surface^64^. The associated grazing trails in the surfaces surrounding the arenicolid mounds most likely record exploitation of microphytobenthos further suggesting the existence of ecologic loops that resulted from the activities of the Jurassic ecosystem engineers.

### The taphonomic window

Preservation of delicate, surficial biogenic structures on the seabed is unusual; the trace-fossil record is markedly biased towards subsurface structures produced by infaunal organisms typically preserved as full reliefs within beds or semireliefs on basal surfaces. However, true substrates (i.e. bedding planes that represent the record of sediment–water or sediment-air interfaces at the time of deposition^65,66^) are preserved if a certain combination of taphonomic conditions is met. In the case of the Lastres Formation, the taphonomic window that allowed preservation of the mounds likely involved the interplay of at least five factors: (1) intense mucus production during bioturbation and stabilization by microbial activity; (2) absence of bulldozers and deep-tier crustacean structures; (3) overall low-hydrodynamic energy; (4) relatively high sedimentation rates and frequent mud blanketing; and (5) early carbonate cementation. Arenicolid fecal strings are known to be formed by compacted sediment enriched with mucus and organic matter promoting the preservation of these fragile structures^62,63,67^. In this scenario, mucus, released by microbes and infauna may have contributed to stabilization of the sandy surface, below a thin biofilm. The local absence of sediment bulldozers and deep-tier burrowing crustaceans in these Jurassic coastal facies was also instrumental to the preservation of the delicate positive relief structures preserved on bedding tops^68,69^. Deep- to mid-tier excavators and bulldozers typically destroy evidence of shallower tiers and surficial structures^70,71^. Although crustacean burrows, such as *Thalassinoides* and *Ophiomorpha*, are common in other intervals of the Lastres Formation, they do not occur in the deposits hosting the mounds. The restricted seaway in which the deposits of the Lastres Formation accumulated has been reconstructed as a temperate, protected embayment (shelf lagoon), with low frequency of storms and separated from the open sea by a tectonic threshold formed during a rifting episode^8,16^. As a result, the coastal region was dominated by low-energy conditions in the absence of strong waves and under a microtidal regime^17^. These deltaic facies preserve multiple bedding planes with extensive dinosaur tracksites^7–9,13,14,16^ and other reptile tracks^10,11^. This is consistent with a sheltered low-energy environment characterized by limited erosion and relatively high sedimentation rates. The rich inventory of invertebrate and vertebrate structures distributed along the Lastres Formation underscores the exceptional conditions for preservation of trace fossils in the Lastres Formation, rather than a particular stratigraphic level. At Arroyo Solero outcrop, fine-grained sediment blanketing is revealed by the preservation of a thin (0.1–0.2 cm) mudstone layer mantling the surface. Mud blanketing is attributed to rapid deposition of a deltaic plume and is deemed to have played a significant role in providing the conditions to cross the taphonomic barrier. Finally, the presence of Fe-calcite cement (in addition to quartz) in sandstone beds suggests that carbonate- and iron-rich fluids, resulting from the partial dissolution of shells from overlying transgressive shell beds and carbonate clasts, may have assisted in early cementation aiding the preservability of the surficial biogenic structures.

## Materials and methods

A stratigraphic section was measured at Arroyo Solero. Sedimentary facies were described, based on lithology, physical sedimentary structures, bed boundaries, bed geometry, and fossil content. Interpretations were made in terms of depositional processes and sedimentary environment. Occurrences of trace fossils through the succession were documented. Density of mounds was measured on bedding planes both in large, collected blocks and in situ on well-exposed surfaces in the field. Samples containing specimens of fecal casts were collected in both localities and are housed at the Museo del Jurásico de Asturias (MUJA). Fossil material was photographed with a Panasonic Lumix DMC-TZ30 camera fitted with an objective LEICA 1:3.3–6.4/4.3 lens. Petrographic thin sections prepared from the fossil samples were produced at both the MUJA and the University of Saskatchewan. Jurassic occurrences were compared with modern ones based on observations at various locales, including the Villaviciosa estuary in Asturias and the Island of Sylt in the German Wadden Sea, as well as in aquaria.

### Supplementary Information

Supplementary Legends.

Supplementary Figure S1.

Supplementary Figure S2.

Supplementary Figure S3.

Supplementary Table S1.

Supplementary Table S2.

## Data Availability

All data generated or analysed during this study are included in this published article (and its supplementary information files).

## Appendix

*Cumulusichnus* igen. nov.

Type ichnospecies: *Cumulusichnus asturiensis*

Etymology: From the Latin “cumulus” meaning heap, mound, or accumulation.

Diagnosis: Curled strands of superimposed coiled, sandy, cylindrical fecal strings forming discrete cap mounds, defining a biogenic pit-and-mound paleotopography.

Remarks: At first sight, *Cumulusichnus* seems morphologically similar to *Lumbricaria* Münster^72^, particularly *L. intestinum* Münster^72^. As in *Cumulusichnus*, *Lumbricaria* consists of strings of fecal castings^73–75^. However, *Lumbricaria* does not display the distinct cap mound morphology of *Cumulusichnus.* Also, *Lumbricaria* commonly shows constrictions^74,75^, which are absent in the fecal casts forming *Cumulusichnus* and displays a wide range of morphologies that differ from the regular morphology of *Cumulusichnus* (see Fig. 5 in^74^). Regarding its mode of occurrence, *Lumbricaria* typically occurs as discrete, isolated specimens, meanwhile *Cumulusichnus* is characterized by gregarious occurrences. *Cumulusichnus* may occur as clusters of closely spaced specimens that display more than one size, recording long-lived populations. In addition, *Lumbricaria* does not show any associated vertical burrow as recorded in *Cumulusichnus*. The vertical burrow associated to *Cumulusichnus* is genetically related to the formation of the mounds, which in turn define a biogenic paleotopography recording the work of an infaunal community. Contrastingly, *Lumbricaria* is regarded as recording the coprolites of epibenthic echinoderms (holothurians) or nektonic organisms, most likely fishes or cephalopods (including nautiloids and ammonites), which are excreted on or close to the sea bottom^75–77^. Morphologic characteristics of *Cumulusichnus*, particularly the well-developed flattened semispherical shape of the fecal casting pile is considered a first-rank ichnotaxobase. The gregarious mode of occurrence, illustrated by relatively densely populated surfaces, is also a key feature. These features reveal the mode of construction of *Cumulusichnus* as fecal extrusions related to the activities of endobenthic organisms rather than deliveries on the sea bottom. In addition, *Lumbricaria* has a calcite composition, including saccocomid ossicles^77^, differing from *Cumulusichnus*. The paleoenvironmental and paleoecologic implications of *Cumulusichnus* and *Lumbricaria* are markedly different. *Lumbricaria* records fecal material formed on the sea bottom, typically implying formation on neritic settings, whereas *Cumulusichnus* is present in a costal setting, the subaqueous delta plain of the Lastres Formation, mimicking the distribution of modern arenicolid worms which are particularly abundant in intertidal and shallow subtidal areas. As *Cumulusichnus*, the ichnogenus *Chomatichnus* shows a mounded morphology^78^. However, *Chomatichnus* lacks the spaghetti-like coiled fecal castings of *Cumulusichnus*. Additionally, and in contrast to the original description, the fecal nature of the mounds in *Chomatichnus* is far from clear. *Medusites* also consists of long and intertwined string-like elements^79^ but differs from *Cumulusichnus* in its phosphatic composition and in the overall morphology of multiple parallel threads, in places splitting up and rejoining^74^. Comparable fecal strings associated to vertical burrows have been recorded in Carboniferous-Permian glacial shelf deposits of India^80^. However, these strings do not form mounds, lacking the biogenic pit-and-mound topography generated by *Cumulusichnus*. Also, in contrast to *Cumulusichnus*, the strings seem to bifurcate distally. These structures were attributed to crustaceans, and compared with crab structures^80^, regardless the absence of brachyurans in the Paleozoic. Further work is needed to unravel the real affinities of these structures.

*Cumulusichnus asturiensis* n. isp.

Figures 2a–g, 3a–d, 4a–k

Etymology: The ichnospecies name refers to its geographic location in Asturias.

Material: Ten slabs containing 67 specimens (34 from Playa España and 33 from Arroyo Solero) of fecal casts collected and housed at the Museo del Jurásico de Asturias (MUJA) (Tables S1 and S2).

Holotype: MUJA-3594-21 (Fig. 4c).

Paratypes: MUJA-3826-8 to 11 (Fig. 4k), MUJA-3594-17 (Fig. 4b), MUJA-3595-28 (Fig. 4e) and MUJA-3596-2 (Fig. 4h).

Diagnosis: *Cumulusichnus* forming flattened semispherical cap mounds.

Description: See main text.

Remarks: Notably, the described mounds display a very regular flattened semispherical morphology. However, some modern examples tend to be more irregular. We use the regular flattened semispherical morphology as a characteristic at ichnospecific rank. By doing this, we leave open the possibility of finding new ichnospecies of *Cumulusichnus* having a more irregular piled-up of castings resulting in a different mound morphology.
