# Supplementary material for: Jurassic paleosurfaces with fecal mounds reveal the last supper of arenicolid worms
Source: Sci Rep. 2024 Jan 6;14:709. doi: 10.1038/s41598-023-51103-2 (PMC10771522; doi:10.1038/s41598-023-51103-2)
Supplement: Supplementary file 1 — Supplementary Legends. [file 41598_2023_51103_MOESM1_ESM.docx]

**Figure S1.** Simplified geologic map of northern Asturias showing location of the specimens studied. Modified from^74^ and drawn by Laura Piñuela using Corel Draw software version 12.

**Figure S2.** Grain size trends and associated trace fossils. **(a-g)** Playa España slabs. **(h,j)** Arroyo Solero slabs. **(a)** Section of one of the sandstone blocks in which the fecal mounds are preserved (top to upright). Note amalgamation of two beds, the lower one of medium-grained sandstone and the upper one of fine-grained sandstone, separated by an intervening erosional surface mantled by flattened mudstone intraclasts. **(b)** Close-up of the surface of slab MUJA-3596 showing the outlets of tiny vertical burrows with diverse morphologies, as seen in cross section views in (e-g). **(c)** Close-up of the surface of slab MUJA-3595 showing the horizontal trail *Archaeonassa fossulata* (red arrows) and small crater-shaped structures (yellow arrows). **(d)** Detail of one of these crater-shaped structures. **(e,f,g)** Vertical sections of a sandstone block showing muddy sand-filled, thin vertical burrows. **(h)** Close-up of the surface of slab MUJA-4615 with an undetermined grazing trail (red arrows) and protrusive *Diplocraterion parallelum* (yellow arrow). **(i)** Protrusive *Diplocraterion parallelum* in cross-section view (same specimen seen on bedding plane in h)*.*

**Figure S3.** Examples of fecal mounds produced by the arenicolid polychaete *Arenicola marina* around the island of Sylt in the German Wadden Sea. **(a)** Seascape of mound and funnels in the intertidal sand flat during low tide. Scale bar is 50 cm long. **(b)** Close-up view showing details of the tidal-flat topography. Scale bar is 10 cm long. **(c)** Same location after a period of several days with calm conditions. Scale bar is 10 cm long. **(d)** As in **c**, but after a high tide with some wave action that formed sand ripples on the intertidal flat. Scale bar is 10 cm long. **(e)** Close-up view of various casts with fecal strings of varying diameters that correspond to worms of various size classes. Scale bar is 10 cm long. **(f)** Close-up view of a single mound. Scale bar is 1 cm long. **(g)** Close-up views of the lugworm J-shaped burrow from “ant-farm” experiments. Note the mucus lining of the tail shaft. Scale bar is 10 cm long. **(h)** Lugworm J-shaped burrow showing oxygenated (brownish) halo around the gallery and the feeding pocket. Scale bar is 5 cm long.

**Table S1**. Measurements from specimens collected in Playa España.

**Table S2**. Measurements from specimens collected in Arroyo Solero.
