# Supplementary figures and images for: Jurassic paleosurfaces with fecal mounds reveal the last supper of arenicolid worms
Source: Sci Rep. 2024 Jan 6;14:709. doi: 10.1038/s41598-023-51103-2 (PMC10771522; doi:10.1038/s41598-023-51103-2)

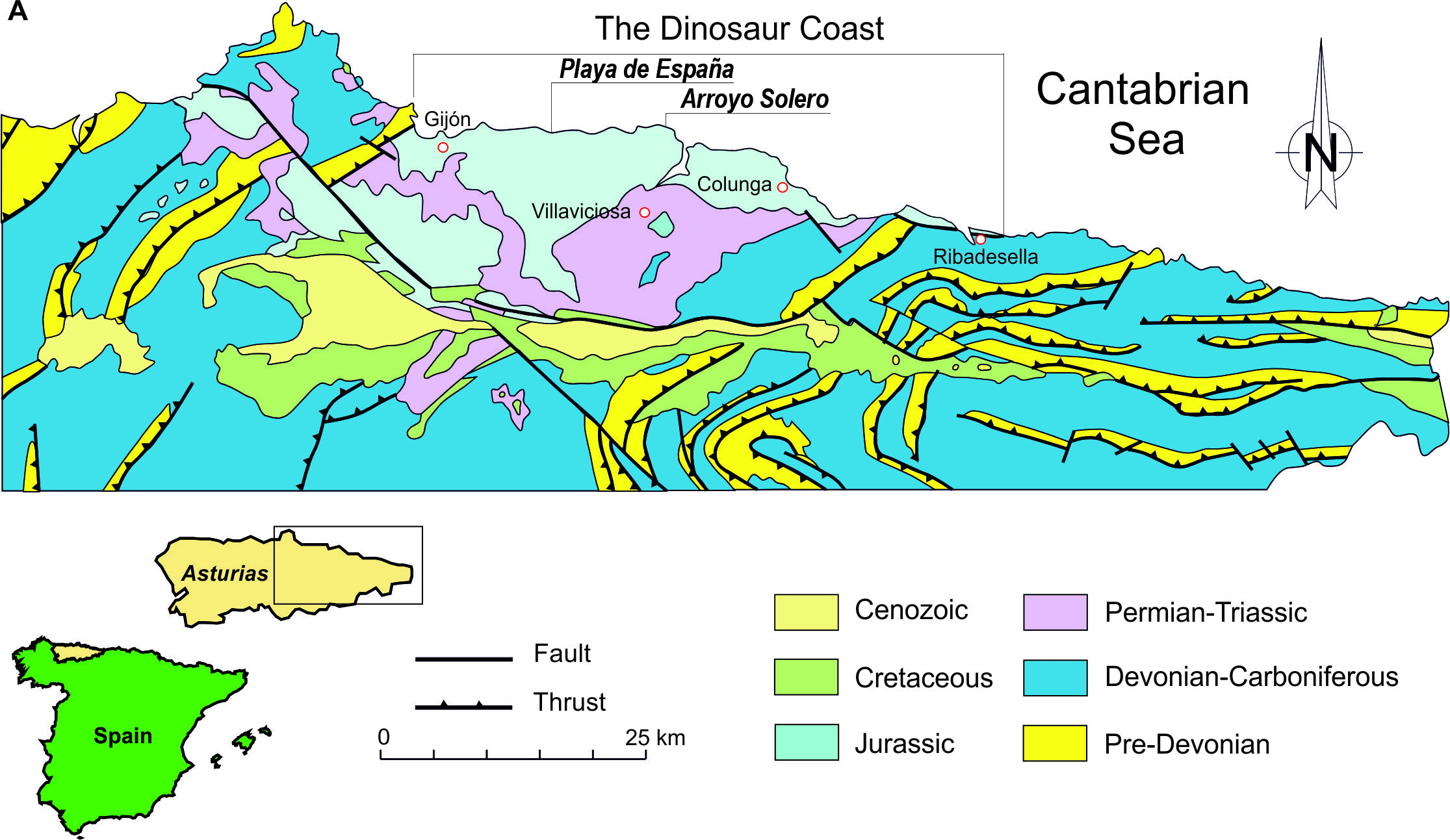

Supplement: Supplementary file 2 — Supplementary Figure S1. [file 41598_2023_51103_MOESM2_ESM.jpg]

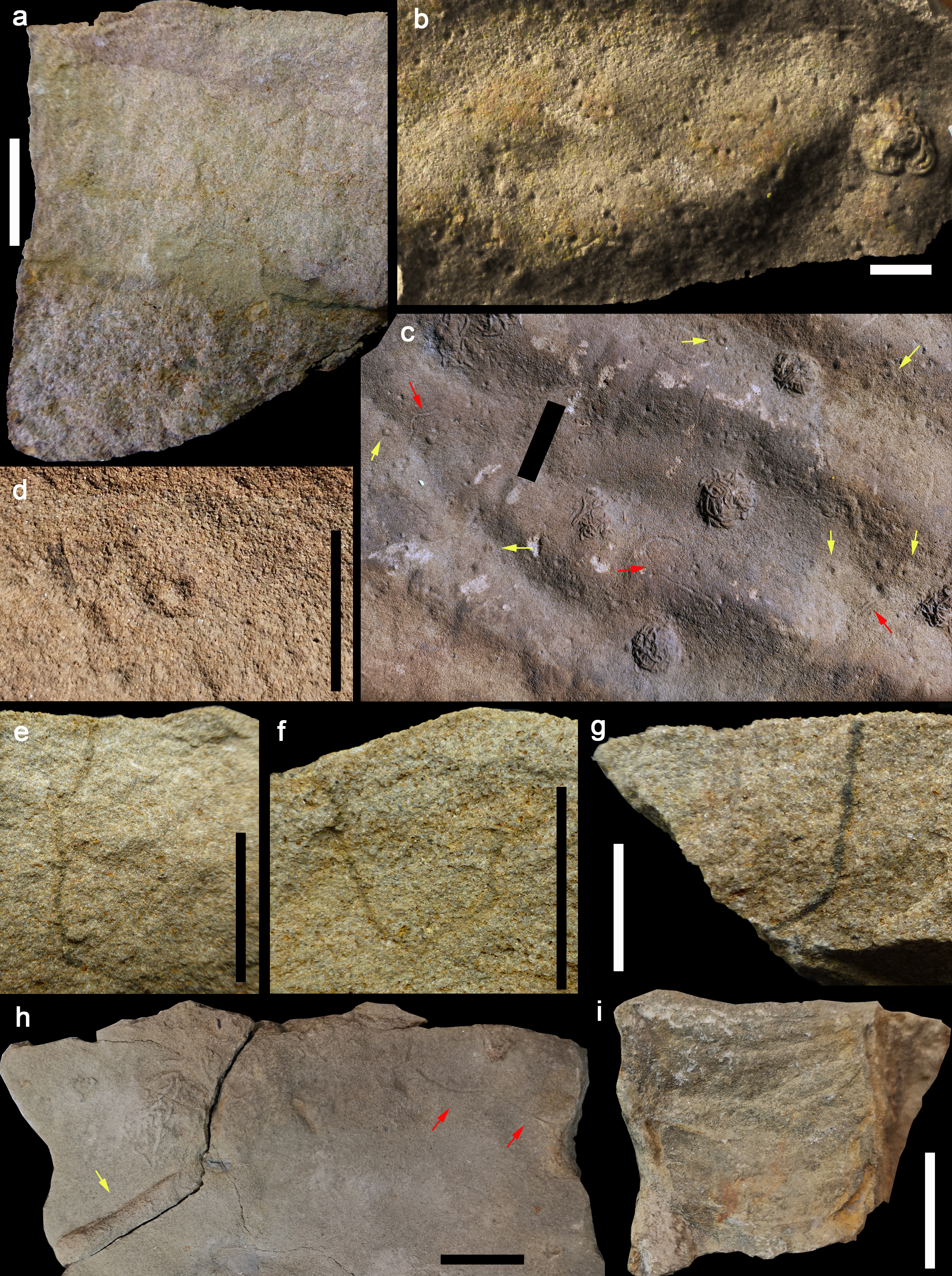

Supplement: Supplementary file 3 — Supplementary Figure S2. [file 41598_2023_51103_MOESM3_ESM.jpg]

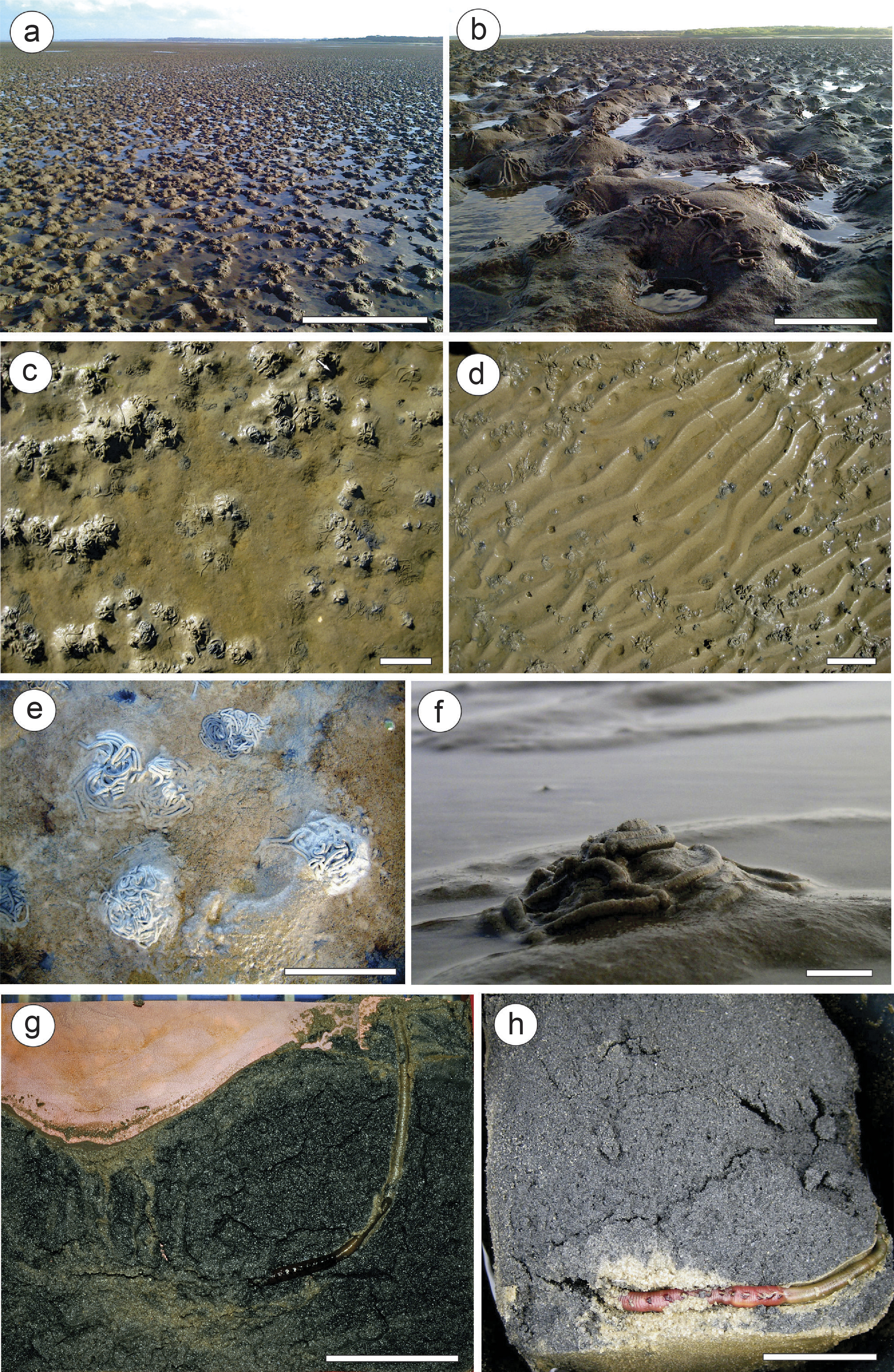

Supplement: Supplementary file 4 — Supplementary Figure S3. [file 41598_2023_51103_MOESM4_ESM.jpg]
